# P-wave Variability and Atrial Fibrillation

**DOI:** 10.1038/srep26799

**Published:** 2016-05-26

**Authors:** Federica Censi, Ivan Corazza, Elisa Reggiani, Giovanni Calcagnini, Eugenio Mattei, Michele Triventi, Giuseppe Boriani

**Affiliations:** 1 Technology and Health Dept., Italian National Institute of Health, Rome, 00161, Italy; 2 Experimental, Diagnostic and Specialty Medicine Department, University of Bologna, Bologna, 40126, Italy; 3 Cardiology Dept., AOU Policlinico di Modena, Modena, 41124, Italy

## Abstract

The analysis of P-wave template has been widely used to extract indices of Atrial Fibrillation (AF) risk stratification. The aim of this paper was to assess the potential of the analysis of the P-wave variability over time in patients suffering from atrial fibrillation. P-wave features extracted from P-wave template together with novel indices of P-wave variability have been estimated in a population of patients suffering from persistent AF and compared to those extracted from control subjects. We quantify the P-wave variability over time using three algorithms and we extracted three novel indices: one based on the cross-correlation coefficients among the P-waves (Cross-Correlation Index, CCI), one associated to variation in amplitude of the P-waves (Amplitude Dispersion Index, ADI), one sensible to the phase shift among P-waves (Warping Index, WI). The control group resulted to be characterized by shorter P-wave duration and by a less amount of fragmentation and variability, respect to AF patients. The parameter CCI shows the highest sensitivity (97.3%) and a good specificity (95%).

The importance of the analysis of the P-wave in the stratification of patient suffering from atrial fibrillation (AF) is worldwide accepted. Nowadays it is recognized that not only the P-wave duration, but also the P-wave morphology has the potential to give information about the anatomical substrate predisposing to AF^1^^,^^2^.

Technically, given the low amplitude of this portion of the ECG signal respect to the background noise, the analysis of the P-wave features have been so far performed on a model of the P-wave, the P-wave template, obtained by the averaging technique.

However, sophisticated ECG systems and some modern electrocardiographs have suitable noise rejection and resolution for ECG acquisition to allow the analysis of the P-wave on a beat-by-beat basis. It is thus important nowadays to attempt studying these never analyzed aspects related to the beat-to-beat depolarization of the atria, to ameliorate the comprehension of arrhythmic phenomena.

To our knowledge, few papers have been published assessing the P-wave variability over time in patients suffering from AF. Recently Martinez and co-authors focused on the time course of some P-wave features with the aim of extracting predictors of AF episode onset^3^^,^^4^.

The aim of this paper was to analyze the potential of P-wave variability for indicating atrial substrate modification correlated with AF. To this aim, P-wave features extracted from P-wave template together with novel indices of P-wave variability have been estimated in a population of patients suffering from persistent AF, and compared to those extracted from control subjects.

## Methods

### ECG acquisition

Study population consisted of 73 patients suffering from persistent AF (43 males, age 69.5 ± 9.3 years) and 20 control subjects (11 males, age 66.8 ± 6.7 years).

Patients suffering from persistent AF were selected among patients underwent electrical cardioversion for nonvalvular persistent AF at the Experimental, Diagnostic and Specialty Medicine Department of the University of Bologna over a 24-month period. Control group was composed by patients without atrial diseases, prior cardiac diseases and history of AF, hospitalized for not cardiovascular factors. Table 1 shows the clinical characteristics of the study population.

ECG has been recorded, after informed consent, in AF patients after the sinus rhythm was restored by electrical cardioversion. ECG signals were acquired using a 16-lead mapping system for high-resolution biopotential measurement (ActiveTwo, Biosemi, The Netherlands), sample frequency 2 kHz, 24 bit resolution, 0–400 Hz bandwidth. A ten minute ECG recording was collected in each subject. 10 out of 16 electrodes were positioned on the thorax to obtained the standard 12-lead ECG.

The diagnosis of sinus rhythm after electrical cardioversion was based on diagnosis made by an expert cardiologist on the basis of 12-lead ECG, as in normal clinical practice.

The protocol foresaw also an ECG recording after 3 and 6 months since cardioversion. The analysis of these data, not reported in this paper, showed that ECG does not change over time in terms of P-wave features.

All clinical investigations were conducted according to Declaration of Helsinki principles. The study was approved by the joint research committee of the Department for Technologies and Health of the National Italian Institute of Health and the Cardiovascular Department of the University of Bologna.

### P-wave extraction and analysis

After extracting the P-waves in a 200 ms-long window (400 samples) starting 300 ms before the corresponding R-wave, a beat-by-beat linear piecewise interpolation was used to remove baseline wander, on each P-wave. Fiducial points for linear interpolation were taken from TP and PQ tracks of each beat. Then, a matrix of the P-waves has been created containing all P-waves except ectopic atrial beats or P-waves with excessive noise or recording artifacts. Exclusion criterion was based on conventional template matching of each P-wave, with a cross-correlation coefficient lower than 0.7 respect to the current template. We empirically found that this value threshold for the cross-correlation coefficient guarantees that only ectopic atrial beats or P-waves with excessive noise or recording artifacts were excluded. Classical time-domain and morphological analysis has been performed on P-wave template^5^,^6^,^7^.

To estimate P-wave variability 3 algorithms have been implemented, based on cross-correlation function, butterfly plots and dynamic time warping.

### P-wave time-domain and morphological analysis

Template extraction by averaging technique has been performed as described in^5^.

P-wave duration has been estimated for each patient and for each lead, using the algorithm described in^5^. For each patient, maximum and minimum P-wave duration in any of the 12 leads (P_max_, P_min_) as well as P wave dispersion (P_disp_ = P_max_ − P_min_) have been extracted.

P-wave morphology has been quantified according to the automatic algorithm recently developed by our group^7^. P-wave morphological analysis was based on a Gaussian fit, i.e. P-wave is modeled by the sum of up to 8 Gaussian functions. The number of Gaussian functions needed to model the P-wave (model order, N), the number of zero crossings (polarity changes, PC) and the sum of relative maxima and minima (Fragmented Conduction Index, FCI) of the model have been defined as morphological parameters of the P-wave. Each morphological parameter has been estimated for each lead. For each patient, the model order averaged over the leads (N_avg_) and the sum of the polarity changes (PC) and of the Fragmented Conduction Index (FCI) obtained for each lead has been considered (PC_sum_, FCI_sum_).

### P-wave variability based on superimposed plots

**Figure 1:**
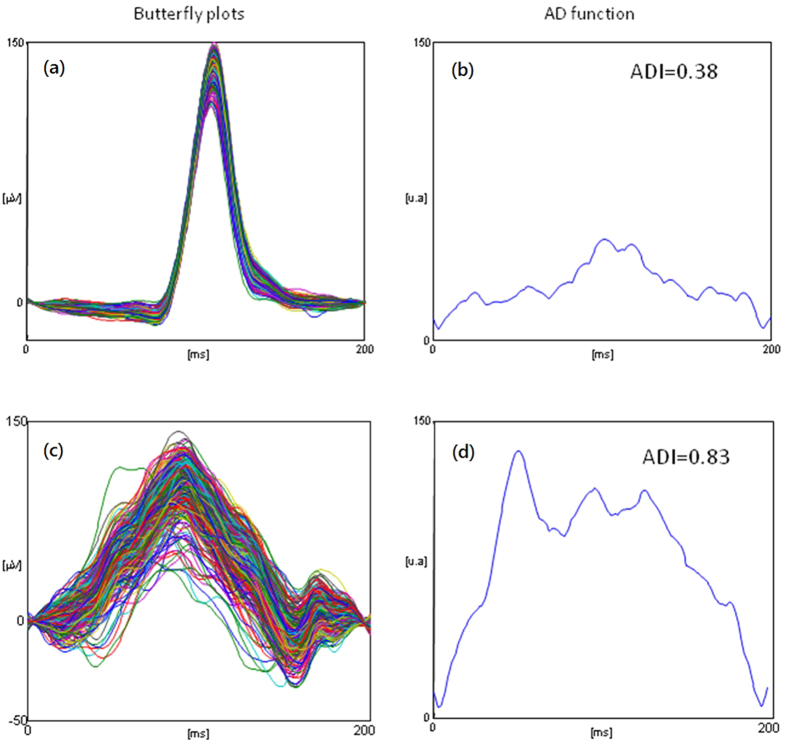
Butterfly plot of P-waves extracted from an healthy subject (**a**) and from a patient with a history of atrial fibrillation (**c**).The time course of the amplitude dispersion are showed in (**b**,**d**), together with the value of amplitude dispersion index (ADI).

The superimposition of the P-waves extracted from an ECG recording (butterfly plot) is a good visual indicator of the variation over time of the morphology of the P-waves. As shown in Fig. 1 (left panels) a butterfly plot of a healthy subject is far different from that of a patient suffering from AF. A quantitative indicator of this difference can be the normalized amplitude difference among any of the P-waves. For each sample, the difference between the maximum value among the P-waves and the minimum value among the P-waves is estimated, obtaining a vector of the amplitude dispersion over time (AD), showed in Fig. 1, right panels. Let P(i, j) be the matrix of the P-waves extracted from an ECG recording of one patient, with i indicating the sample (i = 1,…, 410) and j indicating the j-th P-wave (j = 1,…, number of P-waves in the recording).

Thus the vector AD is obtained as:


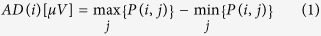


The extracted index of P-wave variability (Amplitude Dispersion Index) is the maximum value of AD divided by the maximum value all over the P-waves (in absolute value):


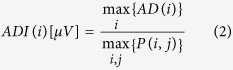


This normalization has been chosen because the maximum value of AD(i) cannot be higher than the maximum value of all P-waves. Thus, for each patient we have 12 values of ADI; as an index of P-wave variability we extracted the mean value of ADI over the 12 leads (ADI_avg_), expressed in arbitrary units (a.u.).

### P-wave variability based on cross-correlation

P-wave variability can be estimated computing the cross-correlation coefficients between the P-wave template extracted from a given ECG recording and each P-wave of the recording that contributed to the P-wave template calculation. An index of P-wave variability can be obtained by averaging the cross-correlation coefficients obtained from the recording (Cross-Correlation Index, CCI). The higher the index, the lower the P-wave variability. As for ADI, from the 12 values of CCI of each patient we extracted the mean value of CCI over the 12 leads (CCI_avg_), expressed in percentage (%).

### P-wave variability based on dynamic time warping

According to the dynamic time warping algorithm the best alignment of waveforms that are similar in shape though out of phase in time can be performed by compressing or extending the time axis of the two signals under comparison. The first step of the algorithm is the computation of the Warping Function (WF) representing the best alignment between the signals.

In this work, a WF has been computed for each pair of P-waves extracted from a recording. Given two P-waves, X and Y:


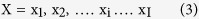



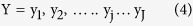


a matrix G (I × J) is built, whose elements g(i, j) represent the relative distance d(x_i_, y_j_) between the two points x_i_ and y_j_ in the corresponding sequences. The WF defines a path in G, through a succession of elements g(i, j), representing the best alignment between X and Y. The path W can thus be expressed as a sequence W of K elements, namely w_k_ = (i, j):


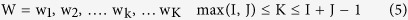


The WF describes the minimum cost path. The WF is made of (I + J)/2 elements and can be obtained by searching in matrix G the minimum cost path leading from g(I, J) to g(1, 1).

In order to quantify the P-wave variability we extracted the warping index (WI), which is the path length, expressed in number of samples. For two identical P-waves, the path length corresponds to the P-wave sample length (that is 410 samples hereby). As for ADI and CCI, from the 12 values of WI we had for each patient we considered the mean value of WI over the 12 leads (WI_avg_).

### Statistical analysis

Comparison between the two classes of patients (persistent AF and control subjects) has been made on the 9 parameters (6 extracted from the template and 3 related to P-wave variability) by non parametric Mann-Whitney U-test for unpaired data. Spearman’s correlation coefficients were used to calculate correlations among the parameters. A value of p < 0.01 has been considered to be significant. Receiver operating characteristic curves were used to evaluate the sensitivities and specificities at different cutoff values of each parameters.

## Results

All AF patients enrolled in this study were successfully cardioverted, and were in sinus rhythm after cardioversion. Wandering pacemaker was diagnosed in no patients. Table 2 shows the results obtained for the AF patients and for the control group. All P-wave indices turned out to be significantly different between the two groups, but P_disp_. As expected the control group is characterized by shorter P-wave duration and less fragmented P-waves. In addition, less P-wave variability over time is associated to control group, as quantified by any of the 3 chosen parameters.

Spearman’s correlation coefficients are reported in Table 3. Values reported in bold indicate correlation higher than 0.5. Pmin and Pmax are correlated and Pmax correlates even with the P-wave variability parameters: the higher the P-wave duration the higher the P-wave variability. The ADI, CCI and WI correlate each other and do not correlate with the morphological parameters.

Table 4 shows the values of sensitivity, specificity, area under curve (AUC) and threshold values for each parameter studied. The parameters with the best sensitivity and specificity turned out to be Pmax, Pmin, FCI and CCI: P_max_ > 121 ms and P_min_ > 97 ms separated patients from controls, with a sensitivity of 95.9% and a specificity of 95%; a FCI > 2.5 separated the two classes with a sensitivity of 89.1% and a specificity of 95%. The P-wave variability parameter CCI showed the highest sensitivity (97.3%) and a good specificity (90%).

If Pmax and CCI are combined, sensitivity and specificity become 98.65 and 100%, respectively. If Navg is also used in combination with Pmax and CCI, sensitivity and specificity both reach 100%.

## Discussion

This study aimed at analyzing the potential of P-wave variability for indicating atrial modifications caused by atrial fibrillation. The link between different P-wave morphologies and different patterns of interatrial conduction in patients with AF has been demonstrated^8^^,^^9^. We quantify the P-wave variability over time using three algorithms and we extracted three novel indices: one based on the cross-correlation coefficients among the P-waves, one associated to variation in amplitude of the P-waves, one sensible to the phase shift among P-waves. In order to investigate the potential of these new indices, we used a population of patients with persistent AF after successful cardioversion compared to subjects without history of AF. More standardized time-domain and morphological P-wave features have also been estimated.

So far the analysis of the P-wave to stratify AF patients and/or to improve the comprehension of the electrophysiological mechanisms of the fibrillating atrium has been carried out on P-wave template^8^^,^^10^^,^^11^^,^^12^^,^^13^^,^^14^. This analysis has been demonstrated to work in extracting indices of AF risk stratification: P-wave duration indices in AF patients resulted to be greater than those in the control patients. Indeed, although it is not completely clear if the atrial substrate is a cause or a consequence of AF, abnormalities in the P-wave reflect structural changes such as increased dispersion of refractoriness, fragmented impulse propagation, atria dilatation and fibrosis which promote slow and inhomogeneous atrial conduction.

However, the use of signal-averaged P wave is not currently in routine use probably because the methods used for P wave analysis vary greatly, and there is an ongoing debate on the appropriate cut-off value for interatrial conduction delay. The conflicting results obtained in literature studies are mainly explained by the different designs, different methodologies, different patient populations and different endpoints^15^.

The two most recent and almost simultaneous investigations on the role of P-wave analysis to predict AF recurrences after external electrical cardioversion used different acquisition systems and different methodologies, had similar population (persistent AF) and found different results^13^^,^^15^. Blanche *et al.* found that none of the P-wave parameters was statistically different between patients with and patients without AF recurrences^15^; Gonna *et al.* reported that parameters associated to P-wave duration were significantly greater in the recurrent AF than in the sinus rhythm group^13^. Both groups concluded that the measurement and use of atrial electrical heterogeneity indices need further research to find accurate and reliable risk factors for AF recurrences in patients with persistent AF.

Thank to the technological advancements in electronics, ECG recording systems now rely on twice the amplitude resolution respect to 10 years ago, consequently providing a higher signal-to-noise ratio. Thus, it is no more mandatory to perform the P-wave analysis on a template, obtained by the averaging technique; it has become possible to perform even a beat-to-beat analysis of the P-wave.

Recently Martinez and co-authors investigated the potential of the quantification of the time course variability of P-wave features in predicting the onset of AF^3^^,^^4^. According to the atrial electrophysiological changes preceding the onset, features like P-wave duration have shown an increasing variability trend, thus suggesting intermittently disturbed atrial conduction in patients close to the onset of AF.

In this paper we confirmed these recent findings, demonstrating that, not only the features extracted from the P-wave, but also the waveform of the P-waves, changes over time in clinical states of AF. Since no wandering pacemaker was diagnosed in any patients, these findings suggest that the atrial impulse path varies on a beat-to-beat basis.

This approach would allow to exploit unknown aspects of the cardiac impulse atrial path in a heart which has already experienced atrial arrhythmias. Atrial conduction disturbance may result in various changes in the atrial activation vector and may lead to variations in P wave durations as well as to abnormal and temporary increases or decreases in P-wave amplitudes.

The control group was selected among patients without atrial diseases, prior cardiac diseases or history of AF. The clinical characteristics of the control group do not differ from those of the persistent AF patients except for atrial arrhythmic diseases. Our analysis revealed electrical atrial path of a AF-damaged heart is far different from that of a healthy heart: in this latter case, the path is the same, beat after beat, whereas in a heart with a substrate damaged by AF, the cardiac impulse passes through different paths, wandering within few beats and even beat after beat. Thus our results reveal the presence of impaired interatrial conduction which may also occur transiently on a beat-to-beat basis, similarly to the interatrial blocks^2^.

The best performing index was the one based on the cross-correlation analysis (CCI), which turned out to have good sensitivity and specificity. In control subjects the cross correlation coefficient is always higher than 0.99, while in AF patients it can reach values as low as 0.8.

The analysis of the P-wave variability should be also considered in the light of the important clinical results obtained from the analysis of the T-wave variability (T-wave alternans) in the stratification of patients at risk of cardiac arrhythmias. Such an analysis was possible since the first nineties, given the best SNR associated to the T-wave, respect to that of the P-wave.

Morphological parameters showed similar sensitivity and specificity obtained in previous papers^6^^,^^7^ and resulted useful to investigate the fibrillating atria when used in combination with time-domain or P-wave variability indices. Although P_disp_ is considered a marker of anisotropic or inhomogeneous atrial conduction, in our population it resulted not to be useful to distinguish patients with persistent AF from controls. As stated before, there are conflicting results in literature concerning the analysis of the P-wave in the study of different forms of AF. Our population of persistent AF patients consists of both patients who experienced AF recurrences after cardioversion and patients who did not. This could explain the higher standard deviation and the poor statistical significance associated to P_disp_ parameter.

It is worth noting that, the ECG resolution needed to detect the morphological P-wave variability is currently available in the most modern electrocardiographs present in the market since about 2 years. Older technologies are not suitable for such analysis, and its diffusion in worldwide healthcare systems is an obstacle for a wide clinical application of the method. Another limitation of the study lies on the small sample size and in the retrospective nature of our study. The potential clinical value of the proposed methods could be further investigated in larger cohort of patients, and in different experimental models which includes paroxysmal or post-operative AF or in screening programs involving subjects aged over 65 years.

Finally, the analysis of the performance of the proposed indices in the prediction of AF recurrences after cardioversion goes beyond the aim of this study, whose objective is to test the reliability of estimating P-wave variability and to investigate its potential clinical application.

In conclusion the quantification of the P-wave variability over time can add information in the understanding of the association between the anatomical atrial substrate and atrial arrhythmias.

## Additional Information

**How to cite this article**: Censi, F. *et al.* P-wave Variability and Atrial Fibrillation. *Sci. Rep.* **6**, 26799; doi: 10.1038/srep26799 (2016).

## Figures and Tables

**Table 1:** Clinical characteristics of AF patients and controls.

	Patients	Controls
	73	20
Gender (male)	43 (59%)	11 (55%)
Age (years)	69.5 ± 9.3	66.8 ± 6.7
Cardiomyopathies	6 (8%)	2 (10%)
Hypertension	50 (68%)	14 (70%)
Diabetes	5 (6%)	1 (5%)
NYHA Class		
I	62 (85%)	20 (100%)
II	8 (11%)	–
III	3 (4%)	–
IV	–	–
LVEF (%)	61%	62%
ACE-inhibitors	22 (30%)	8 (40%)
Sartans	12 (6%)	1 (5%)
Betablockers	53 (73%)	6 (30%)
Diuretics	17 (24%)	4 (20%)
Spironolactone	2 (3%)	v
Ca-channel blockers	8 (11%)	3 (15%)
Nitrates	1 (2%)	–
Digitals	1 (2%)	–
Antiplatelets	6 (8%)	7 (35%)
Anticoagulants	61 (84%)	12 (63%)
Antiarrhythmics	41 (56%)	no
Statine	14 (19%)	3 (15%)

**Table 2:** Values of P-wave indices obtained for AF patients and controls.

	P_max_ (ms)	P_min_ (ms)	P_disp_ (ms)	N_avg_ (#)	FCI_sum_ (#)	PC_sum_ (#)	CCI_avg_ %	ADI_avg_ (a.u.)	WI_avg_ (samples)
AF patients	164.8 ± 21.3	146.4 ± 19.8	18.6 ± 8.7	3.02 ± 0.54	37.0 ± 8.1	9.4±5.9	91.6 ± 4.04	0.98 ± 0.14	636.8 ± 24.6
Controls	87.88 ± 11.6	67.7 ± 11.5	15.9 ± 4.1	2.07±0.33	24.8 ± 4.2	7.7 ± 2.6	99.3 ± 0.53	0.49 ± 0.20	582.9 ± 24.1
p-value	<0.0001	<0.0001	0.06	<0.0001	<0.0001	<0.0001	<0.0001	<0.0001	<0.0001

**Table 3:** Spearman’s correlation coefficients of the p-wave indices.

	P_max_ (ms)	P_min_ (ms)	P_disp_ (ms)	N_avg_ (#)	FCI_sum_ (#)	PC_sum_ (#)	CCI_avg_ %	ADI_avg_ (a.u.)	WI_avg_ (samples)
P_max_ (ms)	1	**0.945**	0.206	**0.645**	**0.624**	0.094	**−0.655**	**0.675**	**0.544**
P_min_ (ms)	**0.945**	1	−0.085	**0.645**	**0.619**	0.058	**−0.601**	**0.620**	0.471
P_disp_ (ms)	0.206	−0.085	1	0.015	0.055	0.076	−0.216	0.230	0.282
N_avg_ (#)	**0.645**	**0.645**	−0.015	1	**0.826**	0.238	−0.461	0.438	0.487
FCI_sum_ (#)	**0.624**	**0.619**	0.055	**0.826**	1	0.252	−0.466	0.444	0.464
PC_sum_ (#)	0.094	0.058	0.076	0.238	0.252	1	−0.126	0.166	0.099
CCI_avg_ %	**−0.655**	**−0.601**	−0.216	−0.461	−0.466	−0.126	1	**−0.879**	**−0.902**
ADI_avg_ (a.u.)	**0.675**	**0.620**	0.230	0.438	0.444	0.166	**−0.879**	1	**0.750**
WI_avg_ (samples)	**0.544**	0.471	0.282	0.487	0.464	0.099	**−0.902**	**0.750**	1

**Table 4:** Values of sensitivity, specificity, area under curve and threshold values for each parameter studied.

	Sensitivity	Specificity	Area under curve	Threshold value
P_max_ (ms)	95.9	95.0	0.94	121.1
P_min_ (ms)	95.9	95.0	0.95	97.2
P_disp_ (ms)	26.1	80.0	0.44	23.9
N_avg (#)_	83.6	95.0	0.88	2.5
FCI_sum (#)_	89.1	95.0	0.84	27
PC_sum (#)_	23.3	90.0	0.50	12
CCI_avg %_	98.6	95.0	0.96	0.98
ADI_avg_ (a.u.)	87.7	95.0	0.94	0.85
WI_avg (samples)_	84.9	95.0	0.88	613.6
